# Publisher Correction: Proton-dynamic therapy following photosensitiser activation by accelerated protons demonstrated through fluorescence and singlet oxygen production

**DOI:** 10.1038/s41467-021-27175-x

**Published:** 2021-11-30

**Authors:** M. Grigalavicius, M. Mastrangelopoulou, K. Berg, D. Arous, M. Ménard, T. Raabe-Henriksen, E. Brondz, S. Siem, A. Görgen, N. F. J. Edin, E. Malinen, T. A. Theodossiou

**Affiliations:** 1 Department of Radiation Biology, Institute for Cancer Research, Radium Hospital, Oslo University Hospital, Montebello, 0379 Oslo Norway; 2 Department of Physics, University of Oslo, Blinderrn, 0315 Oslo Norway; 3 Department of Medical Physics, Oslo University Hospital, Montebello, 0379 Oslo Norway

**Keywords:** Biological fluorescence, Other photonics, Biological physics

Correction to: *Nature Communications* 10.1038/s41467-019-12042-7, published online 04 September 2019.

The original version of this Article contained an error in Fig. 7b, in which the x-axis was incorrectly labelled the same as Fig. 7a. This has been corrected in both the PDF and HTML versions of the Article.

