# Supplementary material for: Proton-dynamic therapy following photosensitiser activation by accelerated protons demonstrated through fluorescence and singlet oxygen production
Source: Nat Commun. 2019 Sep 4;10:3986. doi: 10.1038/s41467-019-12042-7 (PMC6726622; doi:10.1038/s41467-019-12042-7)
Supplement: Supplementary file 1 — Supplementary Information [file 41467_2019_12042_MOESM1_ESM.pdf]

“Proton-dynamic therapy following photosensitiser activation by accelerated protons demonstrated through fluorescence and singlet oxygen production”

Grigalavicius, M., et al.

Supplementary information

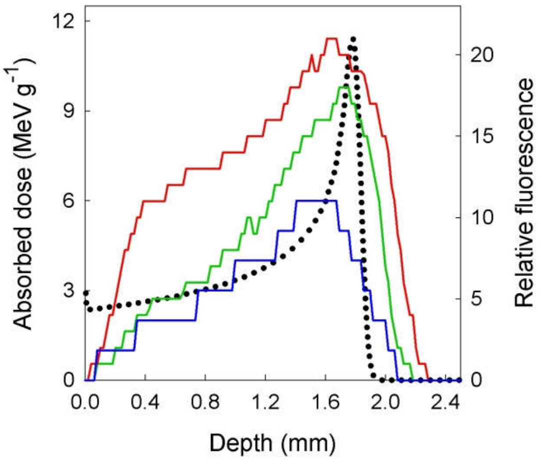

Supplementary Figure 1. Monte Carlo simulated dose deposition of 16 MeV protons in dimethyl sulfoxide (dotted line) vs proton-triggered fluorescence in a solution of mTHPC (55 μM, blue line), protoporphyrin IX (70 μM, green line) and erythrosin B (110 μM, red line).

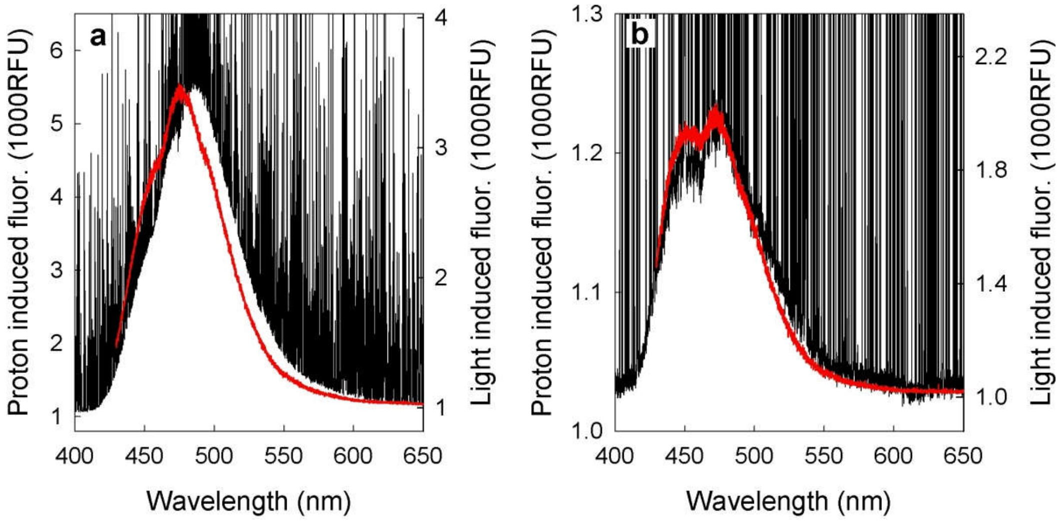

Supplementary Figure 2. Spectral profiles of proton-induced fluorescence in UV dyes (coumarins). Emission spectra for **a** coumarin 102 and **b** 6,8-difluoro-7-hydroxy-4-methylcoumarin obtained under proton excitation are shown in separate panels in black. The two dyes were studied at **a** 200 μM and **b** 5mM concentration in dimethyl sulfoxide. The corresponding spectra of the photosensitisers under light excitation (407 nm) are shown in red.

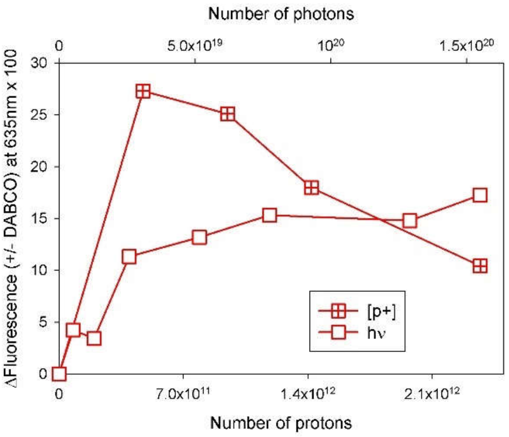

Supplementary Figure 3. Relative protection (%) from fluorescence degradation by singlet oxygen scavenger DABCO. The data were derived from Fig. 6d.

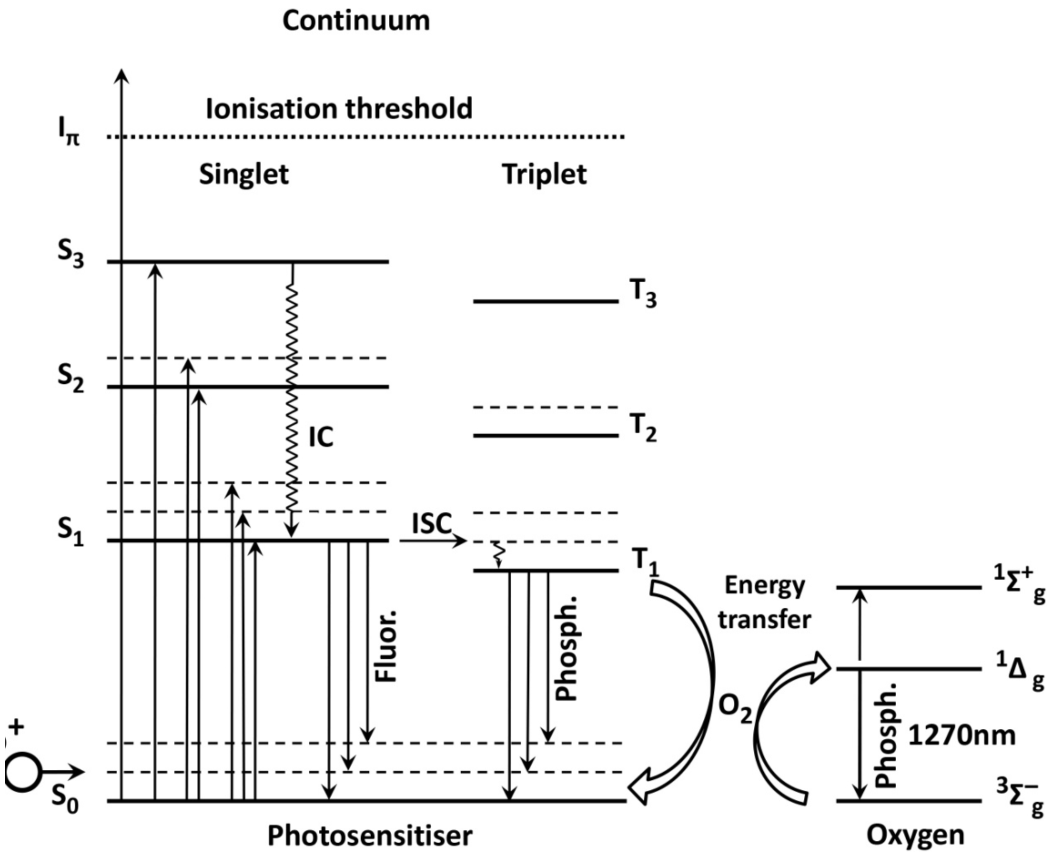

Supplementary Figure 4. A simplified Jablonski scheme describing the main processes in the formation of singlet oxygen ($^1\Delta_g$) from solutions of photosensitisers under proton excitation.

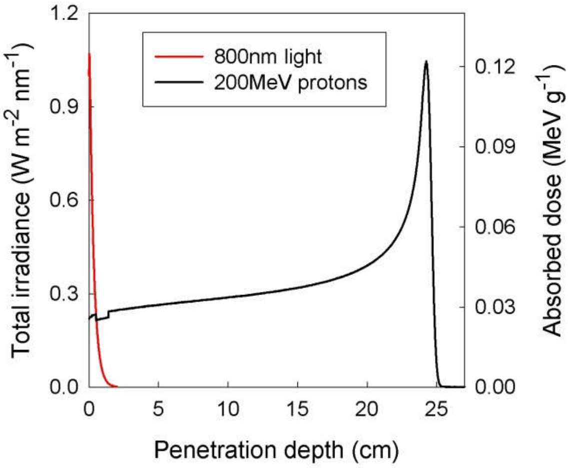

Supplementary Figure 5. Light (800 nm) penetration into skin vs dose deposition of protons (200 MeV) in a head phantom simulating the skin, cranium, and brain.

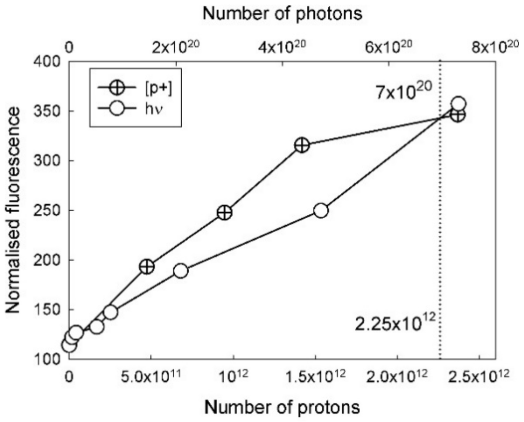

Supplementary Figure 6. Evolution of fluorescence of photo-protoporphyrin IX at 670 nm following proton or photon irradiation, after deconvolution of protoporphyrin IX photobleaching by each time normalising spectra to 635 nm.

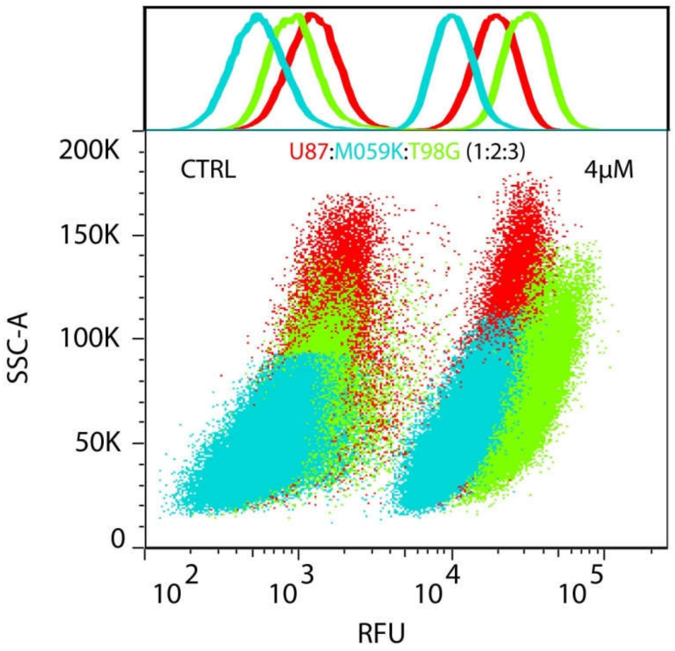

Supplementary Figure 7. Representative dot plots and adjunct histograms of cercosporin uptake by T98G (green), M059K (red), and U87 cells (cyan) in cercosporin (cerco) untreated and treated (4μM, 4h) cells. Uptake was measured by flow cytometry after incubation with cerco.
