# Supplementary material for: Proton-dynamic therapy following photosensitiser activation by accelerated protons demonstrated through fluorescence and singlet oxygen production
Source: Nat Commun. 2019 Sep 4;10:3986. doi: 10.1038/s41467-019-12042-7 (PMC6726622; doi:10.1038/s41467-019-12042-7)
Supplement: Supplementary file 3 — Reporting Summary [file 41467_2019_12042_MOESM3_ESM.pdf]

nature research

Corresponding author(s): *Double-blind peer review submissions: write DBPR and your manuscript number here instead of author names.*

Last updated by author(s): *YYYY-MM-DD*

Reporting Summary

Nature Research wishes to improve the reproducibility of the work that we publish. This form provides structure for consistency and transparency in reporting. For further information on Nature Research policies, see Authors & Referees and the Editorial Policy Checklist.

Statistics

For all statistical analyses, confirm that the following items are present in the figure legend, table legend, main text, or Methods section.

n/a	Confirmed	
☐	☒	The exact sample size (n) for each experimental group/condition, given as a discrete number and unit of measurement
☐	☒	A statement on whether measurements were taken from distinct samples or whether the same sample was measured repeatedly
☐	☒	The statistical test(s) used AND whether they are one- or two-sided<br>*Only common tests should be described solely by name; describe more complex techniques in the Methods section.*
☒	☐	A description of all covariates tested
☒	☐	A description of any assumptions or corrections, such as tests of normality and adjustment for multiple comparisons
☐	☒	A full description of the statistical parameters including central tendency (e.g. means) or other basic estimates (e.g. regression coefficient) AND variation (e.g. standard deviation) or associated estimates of uncertainty (e.g. confidence intervals)
☐	☒	For null hypothesis testing, the test statistic (e.g. F, t, r) with confidence intervals, effect sizes, degrees of freedom and P value noted<br>*Give P values as exact values whenever suitable.*
☒	☐	For Bayesian analysis, information on the choice of priors and Markov chain Monte Carlo settings
☒	☐	For hierarchical and complex designs, identification of the appropriate level for tests and full reporting of outcomes
☒	☐	Estimates of effect sizes (e.g. Cohen's d, Pearson's r), indicating how they were calculated

Our web collection on statistics for biologists contains articles on many of the points above.

Software and code

Policy information about availability of computer code

Data collection	BD FACS DiVa v. 8.0.2, Originlab origin 8pro,
Data analysis	Originlab origin 8pro, SYSTAT sigmaplot 14.0, custom-made matlab code for image analysis, Treestar FlowJo v.7.6.1, Fluka MC, AccuRT

For manuscripts utilizing custom algorithms or software that are central to the research but not yet described in published literature, software must be made available to editors/reviewers. We strongly encourage code deposition in a community repository (e.g. GitHub). See the Nature Research guidelines for submitting code & software for further information.

Data

Policy information about availability of data

All manuscripts must include a data availability statement. This statement should provide the following information, where applicable:

- Accession codes, unique identifiers, or web links for publicly available datasets
- A list of figures that have associated raw data
- A description of any restrictions on data availability

The datasets generated during and/or analysed during the current study are available from the corresponding author on reasonable request.

Field-specific reporting

Please select the one below that is the best fit for your research. If you are not sure, read the appropriate sections before making your selection.

☒ Life sciences ☐ Behavioural & social sciences ☐ Ecological, evolutionary & environmental sciences

For a reference copy of the document with all sections, see nature.com/documents/nr-reporting-summary-flat.pdf

Life sciences study design

All studies must disclose on these points even when the disclosure is negative.

Sample size	Different experiments had different sample sizes. For MTT assays at least 3 parallels were included in the analyses and the experiments were run at least three times.
Data exclusions	In occasions data were excluded as outliers in wells where there was something wrong and the rule was a 50% of more deviation from the trend.
Replication	All attempts at replication were successful.
Randomization	For cell groups randomization is obvious since we are talking about large populations.
Blinding	The MTT assays were conducted by a different person to the one performing the experiments, in blind coding.

Reporting for specific materials, systems and methods

We require information from authors about some types of materials, experimental systems and methods used in many studies. Here, indicate whether each material, system or method listed is relevant to your study. If you are not sure if a list item applies to your research, read the appropriate section before selecting a response.

Materials & experimental systems

n/a	Involved in the study
☒	☐ Antibodies
☐	☒ Eukaryotic cell lines
☒	☐ Palaeontology
☒	☐ Animals and other organisms
☒	☐ Human research participants
☒	☐ Clinical data

Methods

n/a	Involved in the study
☒	☐ ChIP-seq
☐	☒ Flow cytometry
☒	☐ MRI-based neuroimaging

Eukaryotic cell lines

Policy information about cell lines

Cell line source(s)	T98G and M059K were directly purchased by ATCC while U87 were taken from the department biobank, initially from ATCC
Authentication	ATCC authenticated.
Mycoplasma contamination	All cells were tested negative for mycoplasma contamination.
Commonly misidentified lines (See ICLAC register)	Non of the cell lines appear in the ICLAC register

Flow Cytometry

Plots

Confirm that:

☒ The axis labels state the marker and fluorochrome used (e.g. CD4-FITC).

☒ The axis scales are clearly visible. Include numbers along axes only for bottom left plot of group (a 'group' is an analysis of identical markers).

☒ All plots are contour plots with outliers or pseudocolor plots.

☒ A numerical value for number of cells or percentage (with statistics) is provided.

Methodology

Sample preparation	All 3 cell lines subjected to flow cytometric analysis were purchased from ATCC. The cells were incubated with 4 µM cercosporin for 4h and then trypsinised and subjected to flow cytometry analysis. No further tissue processing was involved.
Instrument	The LSRII (BD Biosciences, Franklin Lakes, NJ, USA) cytometer was used for the flow cytometry.

Software	BD FACS DiVa v. 8.0.2 (BD Biosciences, San Jose, CA, USA) software were used to collect the flow cytometry data. The analysis of the collected data was made by using the FlowJo v.7.6.1 software (Treestar Inc., Ashland, OR, USA).
Cell population abundance	No cell sorting was necessary. Pure cell lines purchased from ATCC were subjected to the cercosporin loading experiments. At least 20,000 events were accumulated in each run.
Gating strategy	The starting FSC/SSC gating was based on the intention to cut out obvious cell debris having the weakest signal in the FSC-A and SSC-A channels and to leave only the main population for the further analysis. The additional SSC-A/SSC-W gate eliminated the population of double cells that was parallel to and not within the population of single cells. Within the single cell population, the fluorescence intensity was registered in the PerCP channel (685/35-nm bandpass filter), represented in the X axis, while the Y axis represented the SSC-A signal.

☐ Tick this box to confirm that a figure exemplifying the gating strategy is provided in the Supplementary Information.
